# Endogenous Endophthalmitis: A Review of Case Series Published between 2011 and 2020

**DOI:** 10.1155/2020/8869590

**Published:** 2020-10-23

**Authors:** Ciprian Danielescu, Nicoleta Anton, Horia Tudor Stanca, Mihnea Munteanu

**Affiliations:** ^1^Department of Ophthalmology, “Gr. T. Popa” University of Medicine and Pharmacy, Iasi 700111, Romania; ^2^Department of Ophthalmology, “Carol Davila” University of Medicine and Pharmacy, Bucharest 020021, Romania; ^3^Department of Ophthalmology, “Victor Babes” University of Medicine and Pharmacy, Timisoara 300041, Romania

## Abstract

This is a literature review of 31 case series of endogenous endophthalmitis (EE) published in the last ten years, identified from a literature search of several databases (PubMed, EMBASE, and the Cochrane Library). While diabetes mellitus and malignancies remain the most frequently associated medical conditions, intravenous drug use is a significant risk factor (especially in the last years, in studies from Western countries). Ophthalmologic screening is recommended for candidaemia, but not in patients with sepsis of other aetiologies (however, the physician treating patients with sepsis must be well aware of EE). The most frequent Gram-positive microorganisms that cause EE are *Staphylococcus* and *Streptococcus*; the most frequent Gram-negative organism is *Pseudomonas*, and yeasts, probably *Candida*, usually cause fungal infections. In all-cause EE, prognostic factors of better visual outcomes are initial VA better than counting fingers, performing a pars plana vitrectomy (PPV), performing an intravitreal injection within the first 24 hours after clinical diagnosis, and the presence of a focal type of EE. In endogenous fungal endophthalmitis, more than 1/4 of patients have bilateral involvement. Blood samples have a low rate of positivity. Yeasts remain the most prevalent cause. Many authors report using azoles and echinocandins for systemic therapy (and voriconazole for intravitreal injections). Although PPV was performed in small proportions of eyes, the anatomical success rate is quite high. *Klebsiella pneumoniae* is an important cause of EE in Southeast Asia (and probably an emergent etiology in other regions), which is frequently associated with diabetes. There is a robust association with pyogenic liver abscess (PLA) (but in up to half of the cases, the diagnosis of EE precedes that of PLA). Blood cultures have a high diagnostic yield, while vitreous samples have a low yield. *K. pneumoniae* may carry antibiotic resistance. Anatomical and functional success rates are small, but they may be improved with PPV.

## 1. Introduction

Endogenous endophthalmitis (EE) is an intraocular infection caused by hematogenous spread from distant foci. It is an uncommon but visual severe loss cause that may have devastating ocular and systemic complications [1]. Most authors report that EE accounts for 2–8% of all endophthalmitis cases [1–4].

Ocular inflammation may be the first patient complaint, or EE may complicate an already-diagnosed (and treated) systemic infection.

Since EE is relatively rare, there are no guidelines for its treatment. In many cases, collaboration with a medical team (including intensive care and infectious disease specialists) is mandatory. In some cases, it is associated with septicemia and, unlike most ocular inflammations, may have a significant mortality rate. Therefore, it remains a challenge for the ophthalmologist.

Several systematic reviews have been published on this subject 3–5 years ago. We have attempted to study the last decade's literature, focusing on case series, hoping to bring together significant information about the evolution of diagnosis, management, and EE prognosis. Our rationale was that case reports tend to be published if they are unusual, but case series provide valuable insight into ophthalmology departments' real-life experience.

## 2. Methods

A literature search of several databases (PubMed, EMBASE, and the Cochrane Library) was performed using the keyword “endogenous endophthalmitis” and a publication date between 2011 and 2020; articles published in English, French, and German were included. Of the 547 results, we have selected the papers reporting case series of more than 10 eyes and presented the gathered information in tables. Thirty-one series have been found in the literature published between 2011 and 2020, from different aetiologies.  Table 1 presents the information collected from 21 papers reporting 1202 eyes (1020 patients) with EE (17.8% bilateral cases). We have decided to study the specific causes separately (as other studies did) :

  Articles on endogenous fungal endophthalmitis (EFE) are summarized in Table 2 (6 papers including 229 eyes of 180 patients, 27.2% bilateral cases).
  Articles on endogenous *Klebsiella* endophthalmitis (EKE) are presented in Table 3 (4 papers, including 113 eyes of 94 patients, 20.2% bilateral cases). While 2 papers reported cases from Australia and the USA, 70% to 100% of patients were of Asian ethnicity.

## 3. Predisposing Medical Conditions

The list of known risk factors includes the presence of long-term indwelling catheters, intravenous drug abuse (IVDA), chronic immunosuppression (cancer, acquired immunodeficiency syndrome, and organ transplants), debilitating diseases (diabetes mellitus, renal failure, and liver cirrhosis), endocarditis, or urinary tract infections [1, 5–7]. Typically, risk factors such as indwelling urinary, intravenous catheters or systemic immunosuppression are reported in studies published in Western countries. In contrast, uncontrolled diabetes mellitus and hepatobiliary diseases are reported from Southeast Asia [8–11].

Diabetes mellitus was a predisposing medical condition in 19 of the 21 papers reporting EE of various aetiologies (in 9.3% to 85.7% of patients) (Table 1). Malignancies were also reported in 11.7% to 33% of patients. In studies published in Western countries between 2017 and 2020, IVDA was the leading risk factor, presented in 8.3% to 43.3% of patients [12–14]. There was also a paper reporting 30 patients in which all cases were drug abuse-related [15]. Seven authors have reported that recent general surgical procedures had been performed in 6% to 22.7% of patients [7, 8, 13, 16–19].

In a case series of 53 pediatric cases (we have excluded the parasitic causes) reported by Maitray et al., only 13% of patients presented with fever, 3.3% had bronchopneumonia, and 3.3% had diarrhea. The authors attributed the lack of systemic features to the subjects' immunocompromised state (the most likely reason being protein-energy malnutrition) [20].

### 3.1. EFE

Malignancies were present in 21.4% to 69.7% of cases, while IVDA was the predisposing condition in 15.4% to 28.6% of patients (100% of patients in a small series of *Candida* EE) [21] (Table 2). In 3 papers, recent general surgeries were a major predisposing condition, presented in 28.6% to 37.9% of cases [22–24].

In a 22-year retrospective study, patients with EE caused by mold species were significantly more likely to be receiving iatrogenic immunosuppression (including chemotherapy) and have a history of whole-organ transplantation. Also, mold infections were significantly associated with having an indwelling venous line or catheter [23].

In hospitalized patients without a history of IVDA, risk factors for EFE include prolonged hospitalization and parenteral therapy [25, 26].

### 3.2. EKE

Diabetes mellitus was present in all EKE case series, in large proportions of patients (50% to 70%) (Table 3).

## 4. Extraocular Foci of Infection

The presence of endocarditis has been reported in 5.8% to 31% of patients. Seven Asian authors have reported an association with hepatobiliary infections (including pyogenic liver abscess, PLA) in 10% to 39.5%. Others reported that the foci were urinary tract infections (10% to 36% of patients) and indwelling catheters or dialysis vascular access (9.5% to 16%).

### 4.1. EKE

Hepatobiliary infections were the extraocular foci in 77.7% to 100% of patients (except a small study conducted in the USA where 40% of patients had a PLA and 30% had a pulmonary infection) [27].

Endogenous *K. pneumoniae* endophthalmitis appears to be a particularly frequent etiology in East Asia (25% to 60.8% of all EE patients) [11, 27–29]. A high incidence of cholangiohepatitis may be the cause of this predominance [30]. *Klebsiella's* prevalence causing exogenous or endogenous endophthalmitis in non-Asian countries ranges from 3.1% to 5% [27, 31].

EKE is part of an invasive syndrome characterized by multifocal metastatic infection [10, 32, 33]. The incidence of this metastatic infection may be explained by the emergence of *Klebsiella's* virulent strain with hypermucoviscous properties [32, 34]. There is a strong association with PLA (up to 90% of patients with *K. pneumoniae* EE) [32]. *K. pneumoniae* liver abscesses were associated with a 3% to 11% incidence of EE [35]. In a large review by Chen et al., 52.1% of patients were confirmed to have EE before receiving a PLA diagnosis [36].

Diabetes mellitus is a recognized risk factor for *Klebsiella* infection, increasing liver abscesses in patients with the underlying hepatobiliary disease [9]. In patients with liver abscess, diabetes mellitus and the advent of disseminated intravascular coagulation were risk factors for EE [37, 38]. The ability of *Klebsiella* serotypes K1 and K2 to avoid phagocytosis has been demonstrated to be enhanced in diabetes mellitus patients with poor glycaemic control [35].

## 5. Clinical Diagnosis

The presenting symptoms of EE are variable, from mild discomfort and visual loss to severe pain and visual acuity (VA) of light perception or worse [13]. The anterior chamber may present cells, flare, fibrin, or hypopyon. In the posterior segment, clinical signs include vitreous opacification or chorioretinitis. An insidious onset, focal vitreous opacities (“string of pearls”), and chorioretinal infiltrates suggest fungal etiology [1].

Delayed diagnosis or initial misdiagnosis is a common occurrence, reported in 16% to 63% of cases [13]. A large review found initial misdiagnoses in 33% of cases, usually as noninfectious uveitis, conjunctivitis, or orbital cellulitis [5].

## 6. Microbiological Diagnosis

The prevalence of positive blood cultures varied widely from 0 to 100% of cases. Three studies reporting very low rates of positive blood cultures (0 to 3.4%) were conducted in India [7, 8, 20], and Maitray et al. explained this result: all patients had received prior intravenous antibiotics.

The rate of positive vitreous cultures ranged from 18% to 100%. Regan et al. found 28.6% positive results, but when patients with prior antibiotic treatment were excluded, the percentage grew to 41.7% [14]. Ratra et al. reported that ocular fluid samples gave more positive results than blood cultures, probably because all patients with suspected EE immediately underwent an aqueous tap in the outpatient department [7]. Pillai et al. found 80% of vitreous samples positive in fungal cases, but only 50% in bacterial cases [39]. In a large case series where most patients were young and immunocompetent, the rate of positive blood cultures was very low (0.57%) and the rate of positive vitreous cultures was very high (93%) [8].

Modjtahedi et al. reported 69% of vitrectomies with positive cultures (even after initial negative tap and injection) [15]. In a series of 72 patients with endophthalmitis, polymerase chain reaction (PCR) for eubacterial and panfungal genomes demonstrated a 100% rate of microorganism identification and may be considered a gold standard. As there is no statistically significant difference in the results of PCR performed on aqueous humor and vitreous fluid, PCR on aqueous humor could be the method of choice considering the safety and simplicity of its collection [40].

In 7 of 8 studies conducted in Southeast Asia, Gram-negative infections predominated (43.9% to 83.3% of patients) and *K. pneumoniae* was the most frequent microorganism (26.8% to 53% of patients) [6, 12, 17, 30, 41–44]. One exception was a study conducted in Taiwan on chronic dialysis patients, where *Staphylococcus* was the most frequent cause [45]. In two small series from Japan, the prevalent causes were *Staphylococcus* (57%) and *Candida* (30%) [6, 46].

In case series from India, Australia, USA, and Europe, the distribution of the most frequent aetiologies was variable: *Staphylococcus* (4.6% to 62%), *Streptococcus* (0 to 31%), *Pseudomonas* (3.7% to 38.8%), and *Candida* (2% to 32.8%). *Klebsiella* was a rare occurrence (1.5% to 6.2%). One study from Turkey reported 71.2% of *Candida* infections [19].

### 6.1. EFE

In six studies reporting only EFEs, the rate of positive blood cultures ranged from 9.2% to 25.6% (one study reported 90.1%). Only two authors reported the positive vitreous culture rates: 70.7% and 30%, respectively.

Lingappan et al. suggested PPV as the primary diagnostic method (instead of tap) because EE generally begins with seeding the choroid and progresses to the anterior pole [22]. Histopathological studies suggested that *Candida* preferentially sequesters within inflammatory nodules, limiting the yield of culturing techniques; therefore, negative cultures should be interpreted with caution [47].

While many exogenous endophthalmitis cases are caused by molds (Wykoff et al. reported 85%) [48], most EFEs are caused by *Candida sp.* In our study, the predominant microorganisms were yeasts (71.4% to 76.1%), with *Candida* the most frequently found (50% to 65%). The most common mold was *Aspergillus* (11.7% to 16.4%).

### 6.2. EKE

All 4 papers on EKE found high rates of positive blood cultures, from 66.6% to 92.9%. Positive vitreous cultures were reported in 25% to 50% of patients.

## 7. Medical Treatment

There are no specific treatment guidelines for EE [19, 49]. Considering that the path of the microorganism into the eye is by hematogenous spread, intravenous antibiotics/antifungals are used in all cases (in contrast, the guidelines do not support the use of intravenous treatment in endophthalmitis after cataract surgery) [50]. In some instances, systemic therapy was the only therapy administered (32% of chronic dialysis patients in a study) [41, 45]. Only one author reported a low percentage of patients receiving systemic treatment for EFE (58.8% in yeast infections and 87.5% in mold infections) [23].

Initial systemic therapy is empirical, and the recommendations differ significantly: oral ciprofloxacin (750 mg twice a day) [8], systemic vancomycin [14], or intravenous cefotaxime (1 g thrice a day), and intravenous gentamycin (80 mg twice a day) [7]. After receiving the microbiology test results, the treatment is tailored accordingly.

Intravitreal antibiotic/antifungal injections (IVIs) were recommended by all authors in 48% to 100% of eyes (10 papers reported that more than 80% of eyes received IVI). The injections were repeated in 36.7% to 100% of the eyes. In one study, intravitreal injections were repeated, on average, 3.2 times in bacterial infections and 2.5 times in fungal infections [8]. According to the postoperative endophthalmitis guidelines, the most frequently used was the combination of vancomycin and ceftazidime or amikacin [50]. For EFE, the most used intravitreal antifungal was amphotericin B.

Few authors have reported the use of intravitreal corticosteroids. There is likely insufficient support in the current literature to recommend intravitreal corticosteroids as a standard of care [51].

### 7.1. EFE

Intravenous therapy was used in all patients (except for one case series, where 58% of EEs caused by yeasts and 87.5% of those caused by molds received systemic treatment) [23]. The most commonly used antifungals were amphotericin B, fluconazole, voriconazole, caspofungin, or micafungin.

Intravitreal antifungals were administered in 54% to 100% of eyes and were repeated in 33% to 50%. In a small case series of *Candida* endophthalmitis, intravitreal voriconazole (on average, four injections) was associated with a favourable clinical outcome [52]. Newer echinocandin antifungals may be effective against *Candida* resistant to azoles [1, 53].

### 7.2. EKE

All patients received intravenous antibiotics. Intravitreal antibiotics were administered in a large proportion of eyes (84.5% to 100%). Most of the *K. pneumoniae* isolates are susceptible to ampicillin-sulbactam, third-generation cephalosporin, aztreonam, quinolones, and amikacin [32, 36]. Ang et al. (in a study performed in Singapore) have always used intravenous ceftriaxone as first-line therapy (suspecting *Klebsiella* EE) [54]. However, hypervirulent strains may carry extended-spectrum beta-lactamases and carbapenemases [55].

In a retrospective study from Taiwan, most patients received intravitreal injections with a combination of teicoplanin and ceftazidime [44].

## 8. Surgical Treatment

Most authors reported that vitrectomy was performed in a small percentage of eyes (6.5% to 66%) and repeated (as vitreous lavage) in 3.7% to 36.7%. Only 3 papers reported vitrectomies in over 70% of eyes [8, 19, 46]. In one series, vitreous surgery (vitrectomy/lavage) was repeated, on average, 0.2 times in bacterial infections and 2.0 times in fungal infections [8].

### 8.1. EFE

Primary vitrectomies were performed in 24.2% to 56.9% of eyes. One author stated that vitrectomy was indicated only in complications (retinal detachments, macular pucker) [21]. The lowest vitrectomy rate (24.2%) was in a case series that reported a very high mortality rate (52.2%), indicating that probably many patients were too debilitated to be submitted to surgery [56].

### 8.2. EKE

Vitrectomy rates were very low in 8.1% to 26.8% of cases. There was one exception, a small case series that reported vitrectomies performed in 100% of eyes [57].

## 9. Anatomical Success

Anatomical success was defined in most papers as retention of the globe, without intractable retinal detachment or phthisis bulbi. Most authors reported anatomical success rates ranging from 64.3% to 100%. The lowest percentages were recorded in a pediatric case series (47%) and in a series where most patients were young and immunocompetent (43.9%) [8, 20].

### 9.1. EFE

All authors reported high anatomical success rates, from 75% to 100%. In one study, the enucleation rate was significantly higher in EE caused by molds (25%) than yeasts (0%) [23].

### 9.2. EKE

Anatomical success was achieved in 40% to 83.3% of the eyes. Connell et al. have reported a 25% anatomical success in eyes with EKE [16].

## 10. Functional Success

Functional success was usually defined as visual acuity (VA) ≥ 20/400, and it was reported in 4.5% to 64% of cases. One case series of IVDA-related EE reported 75% functional success. Connell et al. reported VA ≥ 20/400 in 35.4% of bacterial cases and 74% of fungal cases [16].

Three studies reported only the percentage of eyes that achieved VA > counting fingers: 32% to 73% [42–44].

### 10.1. EFE

Only three authors reported the functional success: from 33% (in mold infections) to 56% of eyes (in yeast infections) [21, 22, 47]. Patients with EE caused by molds had worse VA at presentation and final follow-up [23].

### 10.2. EKE

Functional success was achieved in 16.6% to 50% of the eyes. The best success (50%) was in a small series where all eyes were subject to vitrectomy [57].

## 11. Mortality Rate

Eleven authors have reported mortality rates (in 9 series, ranging from 3.7% to 21.1%). The mortality rate was 52.2% in a Korean study of fungal endophthalmitis and 38% in a small USA series in which 92% of the identified microorganisms were Gram-positive organisms [56, 58].

## 12. Screening for EE

While EE may be the first manifestation of bacteremia/fungemia, ocular involvement is diagnosed in patients already under treatment for a systemic infection. Therefore, the question has been raised if patients with sepsis should routinely receive an ophthalmological examination.

Vaziri et al., in a large retrospective cross-sectional study on 258092 patients with hematogenous infections and 3704 with fungemia, found an EE incidence rate of 0.05% (the risk was higher in patients with fungemia: 0.4%) [59]. They have also found that leading predictors included infectious meningitis, endocarditis, and abscesses (as infection sources); comorbidities indicative of immunodeficiency (including HIV/AIDS, lymphoma/leukemia, and diabetes with systemic complications); intensive care unit admission; and longer hospital stays. These patient characteristics can help predict patients' risk of developing endogenous endophthalmitis.

Similar results were reported by Wang et al. (in patients with systemic infection, the incidence rate for ophthalmology consultations was 8.4% and 0.3% for endophthalmitis) [60].

Routine ophthalmological screening is not warranted in all sepsis patients.

In a large retrospective cohort study from Taiwan, 0.84% of PLA patients developed EE [61]. In another Korean study, the prevalence of EE in PLA patients was 1.92% (additional risk factors were other systemic infections, abscesses in the right superior segment, and *K. pneumoniae* infection) [62]. A screening ophthalmological examination has been advocated for patients with *K. pneumoniae* sepsis [32].

In patients with candidaemia, several authors have reported the advent of ocular candidiasis (OC) in 16% to 26.5% of cases [63–65]. 50% to 80% of patients with ocular fungal involvement may initially be asymptomatic (or unable to communicate) [66, 67]. Consecutively, current guidelines recommend an ophthalmological examination of all patients with candidaemia [68, 69]. However, more recent studies have reported lower OC rates, from 2.9% to 12.8%, and the necessity for routine ophthalmology consultation has been challenged [70–73].

## 13. Paediatric EE

Paediatric EE accounts for 0.1% to 4% of all cases, the highest incidence being reported from India and the lowest from the USA [20, 74]. We have found only one case series of 53 eyes. The main systemic symptom was fever in 13 % of patients, while 3.3% had pneumonia and 3.3% had diarrhea. There were no positive blood cultures since all patients had received prior intravenous antibiotics, but the rate of positive vitreous cultures was 66.7%. Gram-negative organisms caused seventy-seven percent of cases in children under 5 years. All eyes received intravitreal injections (repeated in 1/3 of cases), and 77% were subjected to PPV. The anatomical success rate was low (47%), and the functional success rate was 30% [20].

## 14. Discussions

The published case reports tend to present an unusual presentation of a disease. In contrast, the case series offers a typical array of aetiologies, courses of the disease, and outcomes (in a specific hospital or region). As Jackson et al. noted, an example is EKE: as its importance has been well established in the literature, cases may be less likely to be reported [5].

When assessing the predisposing medical conditions, we have found that diabetes mellitus was present in 9.3% to 85.7% and malignancies in 11.7% to 33% of patients. Diabetes was especially prevalent in EKE (50% to 70% of patients).

In the case series published in the past 3 years in Western countries, IVDA was an important risk factor, present in 8.3% to 43.3% of patients [12–14]. In a review of 343 EE cases reported between 1986 and 2012, Jackson et al. found an association with IVDA in 5% of cases [5].

The most frequent extraocular foci of infection were endocarditis (5.8% to 31%), urinary tract infections, indwelling catheters, or dialysis vascular access infections. However, in many cases, an extraocular focus was not found. Authors from Asian countries reported a high prevalence of associated hepatobiliary infections linked to EKE.

When a diagnosis of EE is suspected, the clinician should always request blood cultures sampling, which may be positive in 20% to 100%. This is especially helpful when vitreous cultures return negative (however, if the patient is already under parenteral treatment, the results are disappointing, with positive cultures in 0 to 3.4% of patients). Blood cultures have a low yield in EFE (9.2% to 25.6%) and a very high yield in EKE (66.6% to 92.9%). To maximize the rate of positive results, the blood sampling should be performed during fever spikes, before systemic treatment (and three consecutive blood samples should be taken) [14, 18].

The yield of vitreous cultures was highly variable (18% to 100% positive). This rate is higher when no prior antibiotic treatment was administered; thus, ocular fluid sampling should be a high priority for the clinician suspecting an EE diagnosis. In young and immunocompetent patients, the rate of positive vitreous samples was very high (93%).

*Staphylococci* are Gram-positive organisms that grow in pairs, chains, or clusters. Coagulase-negative staphylococci include 11 subspecies, but only *Staph. epidermidis* is consistently pathogenic for humans. Being a prevalent species, that is, colonizing human skin and mucous membranes, it is an increasing aetiology of infections associated with implanted catheters (and the most common cause of postoperative endophthalmitis) [75]. *Staphylococcus aureus* is identified by positive reactions to catalase, coagulase, deoxyribonuclease test, and mannitol fermentation.

*Streptococci* are Gram-positive, catalase-negative, and coagulase-negative cocci that occur in pairs or chains. They are divided into three groups by the type of hemolysis on blood agar: beta-hemolytic (complete lysis of red cells), alpha-hemolytic (green hemolysis), and gamma-hemolytic (no hemolysis). Beta-hemolytic streptococci are characterized as Group A streptococci (*Streptococcus pyogenes*) and Group B streptococci (*Streptococcus agalactiae*) [76].

*Pseudomonas* are Gram-negative, catalase-positive, nonfastidious organisms (thus having a wide distribution in nature) and are predominantly isolated due to nosocomial, opportunistic infections. They are the most common cause of Gram-negative endophthalmitis (in most cases, *P. aeruginosa*, but other species have also been isolated) [75]. Other Gram-negative bacilli found in EE cases are *Haemophilus influenzae* and *Enterobacteriaceae* such as *Escherichia coli* and *Klebsiella*.

Systemic treatment was recommended by all authors given the specific pathogeny of EE, and in some cases, it was the only therapy administered [41, 45]. It should be started immediately after the blood samples for culture have been taken. The used antibiotics varied largely. We believe that the ophthalmologist should consult with an infectious disease specialist and consider the regional prevalence of microorganisms responsible for hematogenous infections. A history of IVDA suggests a fungal or staphylococcal infection. Patients from Western countries with such chronic risk factors as diabetes and malignancies have varied EE aetiologies. The most frequent Gram-positive organisms are *Staphylococcus* and *Streptococcus*; the most frequent Gram-negative organism is *Pseudomonas*, while yeasts, most probably *Candida*, are the usual etiology of fungal infections.

The initial antibiotic treatment should consider general knowledge about the resistance manifested by different species and then modified according to laboratory tests results for antibiotic sensibility (and clinical evolution). *Staph. epidermidis* is often resistant to multiple antibiotics, particularly methicillin (and should be considered resistant to all beta-lactam antibiotics), but almost all strains are sensible to vancomycin and rifampin [75]. *Staph. aureus* usually produces beta-lactamases (with consecutive antimicrobial resistance) [75].

Penicillin is universally active against Group A, B, C, and G streptococci. Amoxicillin and cephalosporins (cephalexin, cefotaxime, and ceftriaxone) are also effective against different streptococcal infections. In allergic patients, the first options are macrolides (clarithromycin) and/or lincosamides (lincomycin and clindamycin) [75, 77].

*Pseudomonas* is usually sensible to aminoglycosides and ceftazidime. Most *K. pneumoniae* isolates are susceptible to ampicillin-sulbactam, third-generation cephalosporin, aztreonam, quinolones, and amikacin [32, 36].

In a 10-year retrospective study, Ratra et al. found that all isolates of *P. aeruginosa* were sensitive to ciprofloxacin, all staphylococci were sensitive to vancomycin, and all *E.coli* isolates were sensitive to amikacin [7]. Jackson et al. stated that intravitreal vancomycin was used in 62% of Gram-positive coverage cases, while ceftazidime was the most commonly used antibiotic for Gram-negative infections (58%) [5]. Recent studies of isolates from endophthalmitis patients found that bacteria in EE cases were most susceptible to levofloxacin, ceftazidime, and cefazolin (however, the authors have not performed a test for vancomycin sensitivity) [78, 79].

*Candida* strains are generally responsive to amphotericin and triazoles [23, 75], but an increasing number of papers report intravenous treatment with antifungals from the echinocandin family.

Intravitreal antibiotics are also recommended by all authors, usually respecting the indications and dosage for postoperative endophthalmitis (many authors have repeated the injections). If administered within 24 hours to supplement immediate systemic antibiotics, they may provide a relatively favorable visual prognosis [58]. The typical intravitreal treatment includes vancomycin and ceftazidime (or vancomycin and amikacin) [14, 16, 36, 39, 42, 54, 58, 75]. In a retrospective study from Taiwan, where *K. pneumoniae* etiology was suspected, most patients received intravitreal injections with a combination of teicoplanin and ceftazidime [44].

Intravitreal antifungals were administered in 54% to 100% of eyes (most frequently amphotericin B, but several authors reported voriconazole) [23]. The intravitreal use of caspofungin, fluconazole, or flucytosine is considered experimental [1].

PPV was performed in a small proportion of eyes (6.5% to 66%). This may be linked to the fact that EE patients have a systemic infection, which is sometimes severe, and patients may not withstand surgery. When PPV is performed, vitreous samples should be sent for microbiological examination, even if previous ocular fluid samples were negative.

The prognosis of EE is worse than in other types of endophthalmitis. In a retrospective study on all-cause endophthalmitis, EE was an independent risk factor for evisceration or enucleation [80].

Globe retention without a phthisis bulbi was reported in 64.3% to 100% of the case series of EE of multiple causes. In EFE, the anatomical success was even higher, from 75% to 100%. The enucleation rate was significantly higher in EE caused by molds (25%) than yeasts (0%) [23].

EKE's prognosis is particularly poor: anatomical success was achieved in 40% to 83.3% of eyes. In a large review (120 eyes with endogenous *K. pneumoniae* endophthalmitis), intravitreal dexamethasone has significantly reduced the need for enucleation (odds ratio = 4) [36].

In a review of eyes with EE caused by Group B *Streptococcus*, Yoshida et al. reported that visual prognosis is poor, 60% of eyes losing all vision [81].

Functional success (defined as final VA ≥ 20/400) was achieved in 4.5% to 64% of the case series with multiple aetiologies. Lim et al. found that EE caused by Gram-negative bacteria had worse visual outcomes than Gram-positive bacteria or fungus EE [30].

In EFE, functional success was reported in 33% to 56% of eyes. Patients with EE caused by molds had worse VA on presentation and final follow-up.

In EE of various aetiologies, factors associated with better visual prognosis were initial VA better than counting fingers (3 studies), PPV (2 studies), focal-type EE (1 study), and intravitreal injection within the first 24 hours after diagnosis (1 study). Ratra et al. found a significantly improved chance for anatomical and functional success with vitrectomy [7]. In a randomized trial performed on bacterial EE cases, silicone oil endotamponade has significantly increased the rate of success at 9 months [82]. In a case series of EFE, VA increased by 2 lines in 25% of eyes after intravitreal injections and in 71% of eyes after vitrectomy plus intravitreal injections [56]. In a large series of 143 eyes, Muda et al. found no difference in visual prognosis between early vitrectomy (within 2 weeks) and delayed vitrectomy [42].

Functional success in EKE was achieved in only 16.6% to 50% of the eyes. Ang et al. found that the risk factors for poor visual outcomes were the presence of hypopyon, unilateral involvement, a longer interval from sepsis onset to ocular symptoms, and the advent of panophtalmia [54]. Chen et. al reported no differences in VA outcomes between eyes with or without early PPV. However, Yoon et al. stated that 6 months after the early PPV for endogenous *K. pneumoniae* endophthalmitis in 50% of eyes, the vision was counting fingers or better [83]. The same results were obtained by Lee et al.: vitrectomy performed within 10 days of the appearance of ocular symptoms resulted in a better visual prognosis than without vitrectomy [29].

Eleven authors have reported mortality rates associated with EE, ranging from 3.7% to 52.2%.

## 15. Conclusions

While diabetes mellitus and malignancies remain the most frequently associated medical conditions, intravenous drug use is a significant risk factor (especially in the last years, in studies from Western countries).

Ophthalmological screening is recommended for candidaemia, but not for patients with sepsis of other causes (however, the physician must be aware of EE's risk).

In all-cause EE, factors of better visual prognosis were as follows: initial VA better than counting fingers, performing a PPV, performing an intravitreal injection in the first 24  hours after clinical diagnosis, and the presence of a focal type of EE.

In endogenous fungal endophthalmitis, more than 1/4 of patients have bilateral involvement. Blood samples have a low rate of positivity. Yeasts remain the most common cause. Many authors report using azoles and echinocandins for systemic therapy (and voriconazole for intravitreal injections). Although PPV was performed in low proportions, anatomical success was quite high. EE caused by molds has worse functional outcomes.

*Klebsiella pneumoniae* is an important cause of EE in Southeast Asia (and probably an emergent etiology in other regions), frequently associated with diabetes. There is an influential association with pyogenic liver abscess (PLA) (but in up to half of the cases, EE's diagnosis precedes that of PLA). Blood cultures have a high diagnostic yield, while vitreous samples have a low yield. Hypervirulent *K. pneumoniae* may carry antibiotic resistance. Anatomical and functional success rates are small, but they may be improved with PPV.

## Figures and Tables

**Table 1:** Case series: endogenous endophthalmitis of various etiologies.

Author	No. of eyes/patients	Country	Gram-positive/Gram-negative/fungi %	Predisposing medical conditions %	Focus of infection %	Positive blood culture %	Positive vitreous culture %	Intravitreal antibiotic/antifungal (and percentage of repeated injections)	Pars plana vitrectomy %	Anatomical success %	Functional success % (defined as final VA >20/400)	Comments
Connell et al. [16]	64/64	Australia	14.6/19.5/65.9 *S. aureus* 4.6 *Klebsiella* 6.2 *Candida* 32.8	IVDU 38 Diabetes 9.3 Surgery (orthopaedic) 6.2	Genitourinary 12 Indwelling catheter 12		64.1	100	57	92.1	35.4 bacterial cases 74 fungal cases	

Chung et al. [41]	18/18	Korea	16.7/83.3/0 *Staphylococcus* 11 *Pseudomonas* 38.8 *Klebsiella* 38.8	Diabetes 50 Liver cirrhosis 16.7	Pneumonia 22.2 Liver abscess 16.7	55.5	61.1	50	50	90.9	4.5	16.7% received only parenteral antibiotic

Yonekawa et al. [58]	18/13	USA	Gram-positive 92 (S. aureus 61 and Streptococcus 31)	Diabetes 38 Hospital-acquired infection 15	Endocarditis 31	85	33	94 (61)		81 (of survivors' eyes)	55	Mortality 38% Intravitreal injection in the first 24 hours is associated with better visual outcomes

Wu et al. [17]	22/21	Hong Kong	22.7/50/27.3 *Klebsiella* 36.3 *Streptococcus* 13.6 *Candida* 22.7	Diabetes 50 Malignancy 18.2 Recent general surgery 22.7	Urinary 36.4 Septicemia 31.8 Liver abscess 18.2	100	18	72.7	18.2	72.7	22.7	72.7% received systemic treatment Mortality 9% by sepsis

Lim et al. [30]	57/43	Korea	28/54.3/17.5 *Streptococcus* 17.5 *Klebsiella* 45.6 *Candida* 15.7	Diabetes 46.5 Liver cirrhosis 20.9	Liver abscess 39.5 Endocarditis 11.6 Candidemia 9.3 Pneumonia 9.3	81.4	33.3		40.3	96.49	38.5 (75.4 >CF)	Initial VA > CF associated with better visual outcome

Nishida et al. [6]	27/21	Japan	76.2/19/0 *Staphylococcus* 57 *Streptococcus* 23.8 *Klebsiella* 9.5	Diabetes 61.9 Malignancy 28.8 Systemic steroids 28.8	Endocarditis 14.3 Pneumonia 9.5 Peritonitis 9.5 Indwelling catheter 9.5	71.4	28.5	55.5	22.2 (3.7)	81.4	64 (>20/200)	38.1% hospital-acquired infections 76.2% referred by other clinical departments Focal type, initial VA >20/200, and diabetes mellitus are associated with better prognosis Mortality 3.7 %

Ratra et al. [7]	61/58	India	26.5/58.8/14.7 *Staphylococcus* 10.4 *P. aeruginosa* 13.8 *Candida* 3.4	Diabetes 24.1 Surgery 15.5 (abdominal surgery 10.3) Enteric fever 5.2		3.4	27.6	100	62.3 (13.1 repeat vitrectomy)	64.3	29.5 (>20/200)	Favourable anatomical outcome 65.7% of culture-positive and 38.5% of culture-negative cases (statistically significant)

Nachiyappan et al. [46]	10/10	Japan	0/60/40 *Klebsiella* 20 *Candida* 30	Diabetes 40	Colon surgery 20 Disseminated vascular coagulation 40				100	90	40	

Modjtahedi et al. [15]	32/30 IVDA-related	USA	15/7/59	IVDA 100			75	96.8	66		75	69% of vitrectomies had positive cultures (after initial tap and injection)

Bjerrum and la Cour [18]	59/50	Denmark	40/4/2 *Streptococcus* 21 *Klebsiella* 4 *Candida* 2	Diabetes 36 Malignancy 26 Postsurgical 6	Cutaneous ulcer 18 Endocarditis 12 Urosepsis/haemodialysis 10	51	43			88	26% VA>20/40	Mortality 15% in the first year

Kuo et al. [45]	25 chronic dialysis patients 31 normal renal function	Taiwan	44/32/4 *Staphylococcus* 36 *Pseudomonas* 16 *Acremonium* 4 40/28/8 *Staphylococcus* 16 *Streptococcus* 16 *Klebsiella* 28 *Candida* 4	Diabetes 72 Hypertension 80 Diabetes 85.7 Hypertension 41.9	Dialysis vascular access infection 16%	32 41.9	52 58.1	48 93.5	8 6.5	80 80.6	8 22.6	32% received only parenteral therapy No patient received only parenteral therapy

Cho et al. [12]	67/60 61/48	USA Korea	44.8/3/34.3 *Staphylococcus* 23 *Klebsiella* 1.5 *Candida* 28.4 21.3/44.3/16.4 *Streptococcus* 9.9 *Klebsiella* 37.7 *Candida* 16.4	IVDA 43.3 Diabetes 22.4 Diabetes 54.2 Hepatitis/cirrhosis 29.2	Fungemia due to IVDA or indwelling catheter 26.7 Endocarditis 7 Liver abscess 33.3 Fungemia due to IVDA or indwelling catheter 6.3	37.1 81.8	49.2 13.2		56.33 of EBE	77.4 of EBE	46.3 of EBE	Final VA of *Klebsiella* EE was worse than of those with another pathogen

Muda et al. [42]	143/120	Malaysia	32.5/50.6/16.9 *S. aureus* 20.5 *Klebsiella* 32.5 *Candida* 9.6	Diabetes 60 Renal failure 16.7 Malignancy 12.5	Urinary 17.5 Pulmonary 15.8 Skin/soft tissue 14.2 Hepatobiliary 10 Infected catheter 9.2	42	22.3	88.7 (59.5)	51.4	88.8	73 (>counting fingers)	

Maling et al. [13]	54/48	United Kingdom	39.5/4.1/10.4 *S. aureus* 20.8	Diabetes 14.5 IVDA 8.3 Recent surgery 6	Endocarditis 8.3 Septic arthritis 8.3	58	23		11			Mortality 4% in the first 6 months

Silpa-Archa et al. [43]	42/36	Thailand	48.8/43.9/7.3 *Klebsiella* 26.8 *Streptococcus* 29.3	Diabetes 30.6	Liver abscess 19.2 Urinary 19.2	36.7	57.9	88	58.5	90.4	32 (>counting fingers)	Final VA ≥ CF associated with better vision and less severe inflammation (<2+) at presentation

Celiker et al. [19]	21/15	Turkey	9.6/4.8/76 *Candida* 71.2	Diabetes 40 Malignancy 33 Recent surgery 6.7	Fungemia 76.2 Urinary tract infection 13.3 Pneumonia 6.7	60	100	66.7	33.3 (28.6 repeat)	100	42.85	

Maitray et al. [20]	53/53 (after excluding parasitic etiologies) pediatric cases	India	36.7/23.3/6 *Staphylococcus* 9.4 *Pseudomonas* 3.7 *Candida* 3.7	Fever 13	Pneumonia 3.3 Diarrhea 3.3	0 (all have received prior intravenous antibiotic)	66.7	100 (36.7)	77	47	30	Vitrectomy was correlated with good functional outcome 77% of cases in children under 5 years were Gram-negative EE

Dave et al. [8]	173/117	India	48/37/15 *S. pneumoniae* 20 *P. aeruginosa* 8.6 *Candida* 4.6	Diabetes 16.2 Surgical conditions 6.5 (post-solid-organ surgery 2.9 and complicated abortion 2.4)	Cellulitis 2.4 Typhoid fever 1.7 Urinary tract infection 1.2	0.57	93.06	100 (100)	89	43.9	26	Most patients were young and immunocompetent Vitreous is the commonest positive sample Odds of favorable functional outcome: 2.28 when primary PPV was done

Regan et al. [14]	40/35	USA	*Staphylococcus* 40	Diabetes 45 Malignancy 31.4 Lung disease 14.3	IVDA 22.9% Port/catheter 14.3%	48.5	28.6% (41.7% if prior antibiotic-treated were excluded)		14.2			

Pillai et al. [39]	41/34	India	38.4/11.5/50 *Staphylococcus* 20.5 *Aspergillus 20.5*	Diabetes 70.7 Chronic liver disease 29.4 Transplant 14.7 Malignancy 11.7	Urinary tract infection 32.3 Sepsis 20.5 Abscess 14.7 Endocarditis 5.8	20.5	63.4 (80% in fungal cases, 50% in bacterial cases)		53.65	87.8	36.6 (>20/200)	Mortality 20.5% (38.5% in EFE)

Hsieh et al. [44]	83/70	Taiwan	14.4/77.1/8.4 *S. aureus* 14.46 *Klebsiella* 53 *Candida* 7.2	Diabetes 63.86 Malignancy 13.2	Abdominal abscess 43.3 Urinary tract infection 12.5			91.5	46.9	92.7	34.9 (>counting fingers)	Preferred intravitreal antibiotic combination: teicoplanin and ceftazidime (mean number: 3) Mortality 10%

**Table 2:** Case series: endogenous fungal endophthalmitis.

Author	No. of eyes/patients	Country	Microbiology %	Predisposing medical conditions %	Focus of infection %	Positive blood culture %	Positive vitreous culture %	Intravitreal antibiotic/antifungal (and percentage of repeated injections)	Pars plana vitrectomy %	Anatomical success %	Functional success % (defined as final VA >20/400)	Comments
Lingappan et al. [22]	65/51	USA	Yeasts 75 Molds 25 *C. albicans* 65 *Aspergillus fumigatus* 11.7	IVDA 17.6 Malignancy 23.5 Diabetes 21.5 Recent surgery 31.3	Endocarditis 23.5	9.2	70.7	76.9 (36.9)	56.9 (primary vitrectomy) 91 (including subsequent vitrectomies)	80 (4.6 enucleated) 15.3 persistent retinal detachment)	56% of yeast infections 33% of mold infections	94.1% received systemic treatment

Sridhar et al. [23]	67/53	USA	Yeasts 76.1 (*Candida* 55.2) Molds 23.9 (*Aspergillus* 16.4)	Recent surgery 30.8 Malignancy 23.1 Diabetes 17.9 IVDA 15.4 Recent surgery 28.6 Malignancy 21.4 Diabetes 28.6 IVDA 28.6		Positive systemic culture 25.6 Positive systemic culture 14.3		80.4 (33.3) 81.3 (50)	56.9 50	100 (30.6 retinal detachments) 75 (12.5 retinal detachments)		Systemic treatment 58.8 Mortality 5.1% Systemic treatment 87.5 (*p* = 0.035) Mortality 7.1%

Wang et al. [21]	24/14 Candida EE	Taiwan	*Candida* 100	Diabetes 57.1 Malignancy 35.7	Indwelling catheter 42.9			54.2 amphotericin B	Vitrectomy only in complications	91.6	41.7 (>20/200)	If initial VA> 0.1, 100% had final VA > 0.1 If initial VA < 0.1, 17.6% had final VA > 0.1

Kim et al. [56]	31/23	Korea	*Candida* 64.7 *Scedosporium* 5.9	Malignancy 69.7 Central line 63.6 Diabetes 27.3	Disseminated infection 33.3 Pneumonia 18.2 Genitourinary 18.2	90.1		72.7	24.2	100		Mortality 52.2% VA improved >2 lines:25% eyes with intravitreal injection and71% eyes with vitrectomy + intravitreal injection

Tirpack et al. [47]	10/10	USA	*Candida* 40	IVDA 100 Hepatitis C 50		10	30	100 (50)	50	100		

Duan et al. [24]	32/29	China	Yeasts 71.4 Molds 28.6	Recent surgery 37.9 (percutaneous nephrolithotomy 17.2)					87.5		51.72	

**Table 3:** Case series: endogenous *Klebsiella* endophthalmitis.

Author	No. of eyes/patients	Country	Predisposing medical conditions %	Focus of infection %	Positive blood culture %	Positive vitreous culture %	Intravitreal antibiotic/antifungal (and percentage of repeated injections)	Pars plana vitrectomy %	Anatomical success %	Functional success % (defined as final VA >20/400)	Comments
Ang et al. [54]	71/61	Singapore	Diabetes 55.7	Hepatobiliary 77.5 Urinary 9.9	92.9	43.6	84.5	26.8	73.2	22.5	Risk factors for poor visual outcome: Presence of hypopyon Unilateral involvement Interval from sepsis onset to ocular symptoms Panophtalmia

Odouard et al. [57]	6/4	Australia	Diabetes 50	Hepatobiliary 100	75	50	100 (100)	100	83.3	50	All patients were of Southeast Asian ethnicity

Chung et al. [32]	24/19	Hong Kong	Diabetes 52.6 Liver cirrhosis 15.8	Sepsis 100 Liver abscess 94.7 Empyema 15.7	94.7	37.5	89.4	15.8	40	16.6	Mortality 21.1%

Shields et al. [27]	12/10	USA	Diabetes 70	Liver abscess 40 Pulmonary 30 Soft tissue 20	66.6	25	100	8.1	58.3	33.3 (42 VA > CF)	30% of patients were not of Asian ethnicity

## Data Availability

No data were used to support this study.
